# Supplementary material for: Machine learning meets maternal health: Uncovering spatial blind spots in antenatal care quality in Bangladesh
Source: PLoS One. 2025 Nov 26;20(11):e0337643. doi: 10.1371/journal.pone.0337643 (PMC12654952; doi:10.1371/journal.pone.0337643)
Supplement: S1 Table — (DOCX) [file pone.0337643.s001.docx]

**S1 Table: Association between outcomes and exposures, p-value**

|  | | Binary ANC Quality | | p-value |
| --- | --- | --- | --- | --- |
|  |  | Low quality | High quality |  |
| Age in 5-year groups | 15-19 | 525(83.1%) | 107(16.9%) | 0.000 |
|  | 20-24 | 1165(77.6%) | 337(22.4%) |  |
|  | 25-29 | 941(73.7%) | 336(26.3%) |  |
|  | 30-34 | 586(73.8%) | 208(26.2%) |  |
|  | 35-39 | 231(72.4%) | 88(27.6%) |  |
|  | 40-44 | 43(75.4%) | 14(24.6%) |  |
|  | 45-49 | 6(100.0%) | 0(0.0%) |  |
| Division | Barishal | 386(76.7%) | 117(23.3%) | 0.000 |
|  | Chattogram | 610(77.3%) | 179(22.7%) |  |
|  | Dhaka | 503(72.8%) | 188(27.2%) |  |
|  | Khulna | 414(76.2%) | 129(23.8%) |  |
|  | Mymensingh | 392(71.1%) | 159(28.9%) |  |
|  | Rajshahi | 358(74.1%) | 125(25.9%) |  |
|  | Rangpur | 438(82.0%) | 96(18.0%) |  |
|  | Sylhet | 396(80.3%) | 97(19.7%) |  |
| Place of residence | Urban | 1043(67.1%) | 511(32.9%) | 0.000 |
|  | Rural | 2454(80.9%) | 579(19.1%) |  |
| Respondent’s educational level | No education | 165(85.9%) | 27(14.1%) | 0.000 |
|  | Primary | 843(85.7%) | 141(14.3%) |  |
|  | Secondary | 1941(78.4%) | 534(21.6%) |  |
|  | Higher | 548(58.5%) | 388(41.5%) |  |
| Religion | Islam | 3196(76.4%) | 988(23.6%) | 0.265 |
|  | Hinduism | 276(74.2%) | 96(25.8%) |  |
|  | Buddhist | 18(90.0%) | 2(10.0%) |  |
|  | Christianity | 7(63.6%) | 4(36.4%) |  |
| Access to mass media | No | 1560(83.2%) | 314(16.8%) | 0.000 |
|  | Yes | 1937(71.4%) | 776(28.6%) |  |
| Wealth index | Poorest | 750(89.0%) | 93(11.0%) | 0.000 |
|  | Poorer | 751(83.2%) | 152(16.8%) |  |
|  | Middle | 731(78.9%) | 195(21.1%) |  |
|  | Richer | 692(72.8%) | 258(27.2%) |  |
|  | Richest | 573(59.4%) | 392(40.6%) |  |
| Received ANC from the respondent's home | No | 3274(76.2%) | 1020(23.8%) | 0.958 |
|  | Yes | 223(76.1%) | 70(23.9%) |  |
| Received ANC from any public health facility | No | 2306(75.6%) | 743(24.4%) | 0.175 |
|  | Yes | 1191(77.4%) | 347(22.6%) |  |
| Received ANC from any private health facility | No | 1437(79.7%) | 367(20.3%) | 0.000 |
|  | Yes | 2060(74.0%) | 723(26.0%) |  |
| Husband/partner's education level | No education | 515(83.6%) | 101(16.4%) | 0.000 |
|  | Primary | 1131(85.5%) | 192(14.5%) |  |
|  | Secondary | 1246(76.3%) | 387(23.7%) |  |
|  | Higher | 605(59.6%) | 410(40.4%) |  |
| Respondent currently working | No | 2749(75.9%) | 874(24.1%) | 0.266 |
|  | Yes | 748(77.6%) | 216(22.4%) |  |
| The person who usually decides on: the respondent's health care | Respondent alone | 303(75.6%) | 98(24.4%) | 0.000 |
|  | Respondent and husband/partner | 2164(74.3%) | 750(25.7%) |  |
|  | Husband/partner alone | 897(81.1%) | 209(18.9%) |  |
|  | Someone else | 122(79.2%) | 32(20.8%) |  |
|  | Other | 11(91.7%) | 1(8.3%) |  |
| No of ANC visits | less than 4 | 2544(100%) | 0(0%) | 0.000 |
|  | 4 or more | 953(46.6%) | 1090(53.4%) |  |
