# Supplementary material for: Machine learning meets maternal health: Uncovering spatial blind spots in antenatal care quality in Bangladesh
Source: PLoS One. 2025 Nov 26;20(11):e0337643. doi: 10.1371/journal.pone.0337643 (PMC12654952; doi:10.1371/journal.pone.0337643)
Supplement: Attachment — Submitted filename: Response to Reviewers.docx [file pone.0337643.s002.docx]

Sukanta Chakraborty

Department of Statistics

University of Chittagong, Bangladesh.

01 October 2025

To

Russell Kabir, PhD

Academic Editor

PLOS ONE

Subject: Regarding revision of Manuscript ID: PONE-D-25-37518 entitled **“Machine Learning Meets Maternal Health: Uncovering Spatial Blind Spots in Antenatal Care Quality in Bangladesh.”**

Dear Sir,

I sincerely thank you and the reviewers for the thoughtful and constructive feedback on my manuscript entitled “Machine Learning Meets Maternal Health: Uncovering Spatial Blind Spots in Antenatal Care Quality in Bangladesh.” (Manuscript ID: PONE-D-25-37518). I have carefully revised the manuscript in response to the comments. Below, I provide a point-by-point response, organized by section, in accordance with the specific comments by the reviewers. The reviewers’ comments are shown in italics, followed by our responses in regular text. Page and line numbers in my response refer to the revised manuscript.

**Response to Reviewer 1:**

Reviewers' Comment by section:

A. ***Abstract***

*• Replace “forecast ANC quality” with “Classify ANC quality” to reflect cross-sectional nature.*

*• Clearly define “high-quality ANC” briefly in the abstract so readers understand the classification criteria.*

*• The policy implications could be stated more concisely.*

**Response:**

1) I replaced “forecast ANC quality” with “Classify ANC quality” in the abstract (line no: 25) and defined “high-quality ANC” in the very 1^st^ line of the abstract. Moreover, I provide concise policy implications.

B. ***Introduction***

*1. Lines 55–62:*

*Global maternal mortality statistics are well presented, but the Bangladesh-specific burden due to inadequate ANC is not quantified. Include national-level evidence linking poor ANC quality to adverse maternal outcomes.*

**Response:** I appreciate this valuable comment from the reviewer. Accordingly, I linked poor ANC quality to adverse maternal outcomes using national-level evidence. The revised text is on line no. 63-68

*2. Line 64 onwards:*

*“High-quality ANC” is mentioned without definition until Methods. Define early in the introduction using BDHS/WHO criteria.*

**Response:** High-quality ANC/Quality ANC is now defined in abstract using BDHS criteria.

*3. Lines 68–74:*

*The paragraph mixes outcomes of high-quality ANC (skilled birth attendance) with components of ANC quality (tests, counseling). Separate for conceptual clarity.*

**Response:** I agree with the reviewer’s concern. I have modified the paragraph to clarify this point. The changes can be found on Lines [77-83].

*4. Lines 85–88:*

*The statement about LMICs having increased coverage but disparities in standards is correct but unsupported here. Provide citations, preferably Bangladesh-specific.*

**Response:** I thank the reviewer for highlighting the need for supporting evidence. I have now added several Bangladesh-specific references to substantiate this statement. These references are now included in the Introduction (Page 4-5, Lines 93-101).

*5. Lines 105–106:*

*Replace “treatment quality” with “ANC quality” for consistency.*

**Response:** I replaced “treatment quality” with “ANC quality” for consistency in line 119.

*6. Line 110:*

*The claim that ML is required to identify women likely to receive inadequate ANC should be softened. Traditional models could also perform this; emphasize ML’s advantages (e.g., non-linearities, interactions).*

**Response:** I thank the reviewer for this suggestion. I have clarified in the manuscript, with appropriate citations, that while traditional regression models can identify women at risk of inadequate ANC, machine learning (ML) offers advantages such as handling high-dimensional data, capturing non-linear relationships, and modeling complex interactions between socioeconomic, demographic, and geographic factors. Moreover, recent studies have shown that ML often outperforms conventional models in predicting maternal and child health outcomes. Accordingly, I now emphasize that ML is particularly useful for identifying “hidden hotspots” where women attend ANC visits but do not receive adequate care. This revision appears in the Introduction section (Page 6, Lines 123–132).

C. **Methods**

*1. Lines 172–174:*

*Replace* *“generate forecasts” with “predict” or “classify.” Avoid implying temporal forecasting.*

**Response:** I replaced “generate forecasts” with “predict” in line no 195.

*2. Statistical vs. ML models:*

*Which statistical model you used? You only mentions about ML models.*

**Response:** I appreciate this helpful comment. In the revised manuscript, I clarified that I used both traditional statistical models and machine learning methods. Specifically, I employed Logistic Regression (LR) along with advanced ML techniques, including Random Forest (RF) and Gradient Boosting Machine (GBM), to predict the risk of low-quality antenatal care (ANC) and to identify key factors linked to these risks. This clarification now appears in the Methods section (Lines 193-196).

*3. Preprocessing:*

*Describe how categorical variables were handled, how missing data were treated, and whether class imbalance (likely given ~24% high-quality ANC) was addressed.*

**Response:** I appreciate the reviewer’s comment. In the revised manuscript, I have clarified the following:

- Categorical variables: All categorical predictors were transformed into factor variables for modeling.
- Missing data: Observations with missing values in the outcome variable (ANC quality) were excluded. Missing values in predictor variables were retained, as exploratory checks indicated that they did not noticeably affect model training or performance; therefore, no imputation was performed.
- Class imbalance: The outcome variable, ANC quality, was binary (High vs. Low), with approximately 24% High and 76% Low in the training set. To address this moderate imbalance, we applied the ROSE (Random Over-Sampling Examples) technique to generate synthetic samples from the minority class while minimally affecting the majority class. Logistic Regression, Random Forest, and Gradient Boosting Machine models were trained on this balanced dataset, and performance was evaluated on the original, unbalanced test set using accuracy, sensitivity, specificity, and ROC-AUC to reflect real-world class distributions.

These clarifications appear in the Methods section (Lines 245-262).

*4. Model tuning:*

*Provide details on cross-validation, tuning ranges, and rationale for chosen hyperparameters in RF and GBM.*

**Response:** I appreciate the reviewer’s valuable suggestion. While cross-validation was conducted to address potential overfitting, it was not reported in the original manuscript. I have now included detailed information on cross-validation, hyperparameter tuning ranges, and rationale for both models:

- **Random Forest (RF):** We tuned two hyperparameters: the number of trees (ntree, 100–500) and the number of variables at each split (mtry, around √p, where p is the number of predictors). We selected ntree = 100 for model stability and computational efficiency, and the mtry value that minimized the out-of-bag (OOB) error.
- **Gradient Boosting Machine (GBM):** The model was trained with n.trees = 100 and interaction depth = 3, using the Bernoulli loss for binary classification. Hyperparameters (n.trees 50–200, interaction depth 1–5, learning rate 0.01–0.1) were tuned via 5-fold cross-validation to optimize ROC-AUC.

These details are now reported in the revised manuscript (Lines 222–227; 236–242).

*5. Feature importance:*

*Clarify that ML importance scores indicate predictive contribution, not causal influence.*

**Response:** I appreciate the reviewer’s comment. I have clarified in the manuscript that two techniques—Random Forest (RF) and Gradient Boosting Machine (GBM)—were used to assess feature importance by retrieving variable importance scores. Importantly, these scores reflect the predictive contribution of each feature to classifying ANC quality, rather than representing causal effects. This clarification appears in the Data analysis section (Page 11, Lines 265-269).

*6. Hotspot analysis:*

*Justify choice of bottom 40% quartile for “low predicted quality” and discuss potential instability of estimates with small cluster sample sizes.*

**Response:** I am grateful for the reviewer’s comment. “Hidden hotspots” were defined as clusters with ≥4 ANC visits but predicted ANC quality in the lowest 40% quartile, chosen to balance sensitivity and specificity—avoiding both missed at-risk clusters and inclusion of moderate-risk areas. I acknowledge that clusters with smaller sample sizes may produce more variable estimates; to address this, I aggregated data at the cluster level, which reduces individual-level variability while providing meaningful spatial interpretation. This clarification appears on Page 12, Lines 278-290.

D. **Results**

*1. Lines 246–269:*

*The distribution section is repetitive and overly dense with numbers. Group results logically (age → region → residence → education → wealth → media → facility → decision-making → visits) and use concise sentences.*

**Response:** I appreciate the reviewer’s suggestion. In the revised manuscript, I have reorganized the distribution section to follow a logical sequence of predictors: age → region → residence → education → wealth → media exposure → facility type → decision-making → ANC visits. Additionally, I have condensed the text to use concise sentences and reduced repetitive numerical details, presenting key findings clearly while maintaining necessary statistical information. These changes appear in the Results and Discussion section (Lines 299–316).

*2. Statistical significance:*

*When stating differences, indicate whether they are statistically significant and provide p-values or CIs.*

**Response:** I appreciate the reviewer highlighting this point. I have revised the manuscript so that all reported differences now indicate statistical significance, with p-values. These updates appear throughout the Results and Discussion section (Lines 337–354).

*3. Figures/Tables:*

*Ensure all figures have self-contained captions and are readable without referring back to the text.*

**Response:** I appreciate the reviewer’s comment. Some of the figure captions have been revised to be fully self-contained, and figures have been formatted for readability without referring back to the text.

E. **Discussion**

*1. Clarify that identified predictors from ML are not causal determinants but predictive markers.*

**Response:** I acknowledge the reviewer’s comment. In the revised manuscript, I have clarified that the variable importance scores from Random Forest (RF) and Gradient Boosting Machine (GBM) models reflect predictive contributions, not causal effects. While variables such as the number of ANC visits, cluster altitude, division, wealth index, and age were most influential for prediction, these should be interpreted as markers of predictive value rather than determinants of ANC quality. This clarification appears in the Results and Discussion sections (Lines 365-385).

*2. Discuss the possibility of unmeasured confounding and the limitations of BDHS cross-sectional design.*

*3. Expand on how hotspot identification can guide targeted interventions and monitoring.*

**Response for 2 &3:** I acknowledge the reviewer’s comment. I have clarified in the manuscript that the cross-sectional design of the BDHS limits causal inference. There is also the possibility of unmeasured confounding, such as provider competence or facility-level resources, which could affect the observed associations. Additional limitations include recall bias and misclassification of ANC service sources. Despite these constraints, hotspot identification enables targeted interventions, efficient resource allocation, and monitoring to improve ANC quality. These clarifications appear in the Results and Discussion section (Lines 470-484).

- Moreover, Reviewers recommended that “Tables that span pages should repeat column headers.” I did it accordingly, and you will find it in the file labeled “Manuscript”.

**Response to Reviewer 2**

*1. Enhance the Introduction and Literature Review*

**Response:** I acknowledge the reviewer’s comment. In the revised manuscript, I have strengthened the Introduction and Literature Review by:

i. Expanding the discussion on antenatal care (ANC) coverage and quality in low- and middle-income countries, with a focus on Bangladesh.
ii. Incorporating recent Bangladesh-specific studies that highlight disparities in ANC utilization and quality.
iii. Emphasizing gaps in existing research that justify the use of machine learning approaches to predict ANC quality and identify high-risk clusters.

These enhancements provide clearer context and rationale for my study and appear in the Introduction sections.

*2. Refine the Methodology Section:*

**Response:** I acknowledge the reviewer’s comment. In the revised manuscript, we have refined the Methodology section by:

1. Providing a clear description of the study design, data source, and sampling strategy.
2. Detailing variable definitions, including outcome and predictor variables, and handling of categorical variables and missing data.
3. Clarifying the machine learning and statistical models used (Logistic Regression, Random Forest, Gradient Boosting Machine), along with cross-validation procedures, hyperparameter tuning, and handling of class imbalance.
4. Explaining the aggregation of cluster-level data for hotspot analysis and the calculation of predictive probabilities.

These refinements improve transparency and reproducibility and appear in the Methods and Data analysis section.

*3. Improve the Results and Discussion*

**Response:** Thanks to the reviewer for highlighting this section. In the revised manuscript, I have improved the Results and Discussion sections by:

a. Reorganizing the Results section to present findings in a logical sequence (age → region → residence → education → wealth → media → facility → decision-making → ANC visits) with concise sentences and reduced repetition.
b. Ensuring that all reported differences include indications of statistical significance, with p-values.
c. Clarifying the interpretation of machine learning outputs, emphasizing predictive contributions rather than causal relationships.
d. Discussing study limitations, including recall bias, misclassification, cross-sectional design, and potential unmeasured confounding.
e. Highlighting policy and programmatic implications based on the identified hotspots of low-quality ANC.

*4. Strengthen the Conclusion*

**Response:** I suggest specific programs or resource allocations like facility-based services through improved staff training and supervision, expanding outreach and health education initiatives, improving infrastructure and accessibility, and developing monitoring tools to track quality improvements for the identified "hidden hotspots" in Rangpur and Sylhet.

Journal Requirements:

1. I made the revised manuscript according to PLOS ONE's style requirements and uploaded 3 files labeled 'Response to Reviewers', 'Revised Manuscript with Track Changes', and 'Manuscript'.

2. Figure 5 in my submission contains map images which is publicly available from [https://data.humdata.org/dataset/geoboundaries-admin-boundaries-for-bangladesh ,](https://data.humdata.org/dataset/geoboundaries-admin-boundaries-for-bangladesh ) which can be used without permission for humanitarian use only. So, there is no need for written permission.

I am deeply grateful to the editor and reviewers for their thoughtful and constructive feedback, which has significantly strengthened my manuscript. I have carefully revised the paper in response to all comments and sincerely hope that the revised version will meet the high standards of PLOS ONE. I respectfully submit it for your kind consideration for publication.

With best regards

Sukanta Chakraborty
